# Polymyxin Combination Therapy and the Use of Serum Bactericidal Titers in the Management of KPC-Producing *Klebsiella pneumoniae* Infections: A Report of 3 Cases

**DOI:** 10.1155/2011/659769

**Published:** 2011-11-28

**Authors:** Eric Gomez, Martha Sanchez, Zonaira Gul, Carl Urban, Noriel Mariano, Robert H. K. Eng, David B. Huang, Tom Chiang

**Affiliations:** ^1^Infectious Disease Division, Department of Medicine, New Jersey Medical School, University of Medicine and Dentistry of New Jersey, Newark, NJ 07103-2714, USA; ^2^Infectious Disease Division, Department of Medicine, New York Hospital Queens, Flushing, NY 11355, USA; ^3^Infectious Disease Division, Department of Medicine, VA New Jersey Health Care System, 385 Tremont Avenue, 111-ID, East Orange, NJ 07018, USA

## Abstract

Management of patients with KPC-harboring Enterobacteriaceae has become a significant and challenging scenario. We report three cases of KPC-producing *Klebsiella pneumoniae* bacteremia that were successfully treated using combination therapy with polymyxin B and other antimicrobials. Serum bactericidal titers were determined and provided additional clinical guidance in the management of such patients.

## 1. Introduction

Traditionally *in vitro* susceptibility testing utilizing the minimal inhibitory concentration (MIC) has been used to guide antimicrobial treatment [1]. However, using this as a tool has led to problems because of failure to detect complex resistance mechanisms using established guidelines. For more difficult-to-treat infections, such as endocarditis, serum bactericidal titers (SBT) have been used to guide therapeutic management and determine antibiotic interactions during therapy [1, 2]. SBT are determined by obtaining serum samples from patients before (trough) and/or after (peak) the administration of the antimicrobial therapy. After serial dilutions of the serum sample, the sample is incubated with the infecting organism to determine the highest dilution with bactericidal activity [1]. Indirectly, this test will assess the antimicrobial susceptibility of the organism (pharmacodynamics) as well as the free antimicrobial serum concentration (pharmacokinetic).

With increasing prevalence of carbapenemase producing Enterobacteriaceae, the antimicrobial management has been challenging, as these organisms also harbor resistance to multiple antimicrobials [3]. Thus, based on clinical laboratory sensitivities, use of fosfomycin, polymyxins, and tigecycline alone or in combination, may be the only antimicrobials left with activity against these organisms [3, 4]. As a result, some have used antimicrobial combination therapy for the theoretical synergistic advantages, in an effort to improve outcomes [5]. Antimicrobial combination therapy against carbapenemase-producing organisms have been evaluated using time to kill assays or checker board; however, no studies have been done using SBT to monitor outcomes. Here we report three cases of patients with serious infections with *Klebsiella pneumoniae* (KP) possessing KPC carbapenemases treated with combination therapy and SBT evaluation.

## 2. Case 1

An 87-year-old man, nursing-home resident, with a history of dementia, benign prostatic hyperplasia, and chronic obstructive pulmonary disease was transferred to the East Orange Veteran's Affairs (EOVA) Hospital for fever, chills, lethargy, and hypotension. On evaluation at EOVA, he appeared confused and disoriented, complaining of generalized weakness and urinary hesitancy. His temperature was 99.1°F orally; blood pressure was 92/57 mmHg, with heart rate of 90 beats per minute and respiratory rate of 21 breath per minute. Physical examination revealed bilateral crackles at the lung bases. Laboratory results showed a white blood cell (WBC) count of 14.3 × 10^9^/L with 78.8% neutrophils. a Foley catheter was placed and a urinalysis showed 50–70 WBCs, 30–50 red blood cells per high power field (HPF), and many bacteria. Initial chest radiograph (CXR) revealed a new small right medial basilar opacity that was suspicious for subsegmental atelectasis. Levofloxacin was started for suspected health-care associated pneumonia and after intravenous hydration a repeat CXR did not show any evidence of an infectious process. Despite antibiotic therapy, patient continued to have fever of 101.4°F and one out of two sets of blood cultures and urine culture grew multidrug-resistant *Klebsiella pneumoniae*. Levofloxacin was changed to doripenem 1 gram IV every eight hours and polymyxin B 500,000 IV units every 12 hours to treat a complicated urinary tract infection with secondary bacteremia. Repeat blood cultures after two days on the new antibiotic regimen were negative and his fever and leukocytosis resolved. He completed a 14-day course of doripenem and polymyxin B without any reported adverse event and was transferred back to the extended care facility in a stable condition.

## 3. Case 2

A 60-year-old man with past medical history of seizure disorder, ischemic cerebrovascular event, atrial fibrillation, hypertension, coronary artery disease, and diabetes was transferred from a long-term care facility to the EOVA Hospital after suffering witnessed tonic-clonic seizures. EEG showed nonconvulsive status epilepticus. He required endotracheal intubation and intravenous fosphenytoin, propofol and phenobarbital were started but his EEG continued to show status epilepticus. His hospital course was complicated by *Clostridium difficile* diarrhea and septic shock. He was empirically started on vancomycin, piperacillin/tazobactam, and metronidazole. A CXR showed a right lower infiltrate and along with purulent sputum he was diagnosed with a ventilator associated pneumonia. His sputum and blood culture grew multidrug-resistant *Klebsiella pneumoniae* and his antibiotic regimen was changed to tigecycline 50 mg IV every 12 hours and polymyxin B 600,000 IV units every 12 hours. With the new antimicrobial regimen his fever resolved and his repeat blood cultures remained negative. His liver and kidney function tests were stable during his 10-day course of antimicrobials. Due to the lack of improvement of his mental status a brain CT was done and revealed acute ischemic changes in the territories of the left middle cerebral artery and left posterior cerebral artery. The palliative care team discussed the patient's poor prognosis with his family and the decision was made to withdraw all acute medical care. He was terminally extubated, placed on comfort care protocol, and died within 24 hours.

## 4. Case 3

A 66-year-old man with past medical history of atrial fibrillation and hypertension presented to the emergency room of the EOVA with a two-day history of shortness of breath. He was recently hospitalized for right-sided hemicolectomy due to a tubular adenoma in the cecum. On physical examination he had a temperature of 99.4°F, heart rate of 100 beats per minute, respiratory rate of 20 breath per minute, and oxygen saturation on 97% on 2 liters nasal cannula. Breath sounds were decreased on the right lower lung field and his abdomen revealed a well-healed surgical scar without any surrounding erythema. Laboratory workup showed a WCB of 12.3 × 10^9^/L, with 83% neutrophils. A portable CXR showed a large right-sided pleural effusion that was drained through a chest tube. Pleural fluid analysis was consistent with a transudate and pleural fluid cultures were negative. Three days later, the patient had a temperature of 101.8°F with complains of dysuria, his white blood cell count increased to 14.4 × 10^9^/L and urine analysis showed 30–49 WBC/HPF. Of note, a Foley catheter had been placed on admission but was removed 48 hours later. Ciprofloxacin was empirically started for urinary tract infection; however, he continued to have fevers up to 102°F, was tachycardic, and had persistent dysuria. Urine culture and blood cultures grew multidrug-resistant *Klebsiella pneumoniae*. Ciprofloxacin was changed to doripenem 1 gram IV every 12 hours, (infused over 3 hours), rifampin 300 mg orally every 12 hours and polymyxin B 250,000 units IV every 12 hours adjusted for a creatinine clearance of 42 mL/min. Soon after this antibiotic combination was started, the fever and leukocytosis resolved and repeat blood and urine cultures were negative. Kidney and liver function tests were stable during the 14-day course of antimicrobials.

## 5. Methods

Blood culture isolates of KP were collected from the clinical microbiology laboratory and tested for SBT. All KP blood isolates were resistant to amikacin, gentamicin, ciprofloxacin, levofloxacin, imipenem, meropenem, ertapenem, cefepime, piperacillin-tazobactam, trimethoprim-sulfamethoxazole, and ceftriaxone and susceptible to tigecycline and polymyxin B by *E*-test (AB bioMeriéux, Marcy l'Etoile, France). All isolates were *bla*_KPC_ positive by PCR. Serum samples for SBT were collected after 24 hours of antimicrobial treatment. Serum collected 30 minutes after the last antibiotic infusion and 30 minutes before the next antibiotic administration were used for the peak and trough SBT, respectively. Serum samples were frozen at −80°C and SBT testing was performed at New York Hospital Queens (NYHQ) in the Infectious Diseases Research Laboratory. SBT was performed according to the standardized microdilution method using cation-adjusted Mueller-Hinton broth as diluent and 10^5^ colony-forming units/mL as inoculums [6]. The test is performed by serially diluting (twofold) the patient serum and inoculating it with a known concentration of the infecting organism isolated from the same patient. After a 20-hour incubation, the inhibitory titer (SIT) is determined by visually examining the well with the highest dilution showing no growth of the organism. A specified volume is then subcultured from each tube that shows no growth. The plates are then incubated overnight and the resulting colonies are counted. The serum bactericidal titer is the highest dilution for which a defined percentage (99.9%) of killing of the original inoculum can be demonstrated. The turnaround time for this test is approximately 48 hours. *Escherichia coli* ATCC 25922, two well-characterized KP isolates from NYHQ with decreased polymyxin B susceptibility, and a KP ATCC 700603 (SHV-18 positive) were also used to assess the bactericidal and inhibitory activity of the polymyxin B combination therapy. All SBT tests were done in duplicate for consistency. 

## 6. Results

Although an absolute SBT has not been clearly established, most studies have found that SBT of at least 1 : 4 and 1 : 8 are needed to ensure a successful outcome [1]. Serum containing combination therapy with polymyxin B and doripenem was bactericidal against the patient isolate in case 1 with a peak SBT of 1 : 32 (Table 1). However, against an NYQH KP control isolate with decreased polymyxin susceptibility, the peak SBT was only 1 : 4. Although the NYQH KP control isolate showed a lower peak SBT than the patient isolate, there was bactericidal activity throughout the dosing interval (trough SBT of 1 : 4) despite the resistance to polymyxin and doripenem. The higher peak/trough SBT seen against the KP SHV-18 isolate (doripenem and polymyxin susceptible) could be explained by the combined activity of doripenem and polymyxin. In case 2, serum containing polymyxin B and tigecycline combination therapy was bactericidal with a peak SBT 1 : 32. Combination therapy with this serum was not tested against isolates with decreased polymyxin B susceptibility due to insufficient patient serum. In case 3, combination therapy was bactericidal with a peak SBT of 1 : 8 for both, the patient isolate and an NYQH KP isolate with decreased polymyxin susceptibility. The patient isolate in case 3 had decreased susceptibility to polymyxin B and doripenem with an MIC of 3 *μ*g/mL and >32 *μ*g/mL, respectively. In all patients, the trough levels were ≥1 : 4 which indicates that bactericidal activity was maintained throughout the dosing regimen.

## 7. Discussion

KPC-producing KP has been consistently resistant to multiple antimicrobials, being generally susceptible to polymyxins, tigecycline, fosfomycin, and variable susceptibility to aminoglycosides [3]. As a result, polymyxins now have an increasing role in the treatment of these infections. Even though limited clinical studies have failed to demonstrate that polymyxin combination therapy is more effective than polymyxin monotherapy, many clinicians have combined polymyxins with other agents for synergy and concerns of resistance development during therapy [7–9]. In a literature review of patients (*n* = 18) treated with polymyxins, combination therapy had a higher success rate than the monotherapy (73% versus 14%) [3]. The authors suggest that the higher failure rate of polymyxin monotherapy could be due to the development of resistance during therapy. A study by Thiolas et al. suggests the use of colistin with imipenem to circumvent the successive emergence of resistance on monotherapy with each agent [10]. Lee et al. reported decreased polymyxin B susceptibility in 20% of patients with KPC-positive KP infections treated with polymyxin B monotherapy (*n* = 12) but combination therapy with polymyxin B and tigecycline prevented the emergence of resistance to either antibiotic [11]. 

With escalating resistance, clinicians are utilizing more antimicrobial combination regimens for treatment of KPC-producing KP infections [5]. In a study of antibiotic combination therapy for KPC-producing *Enterobacteriaceae*, polymyxins were found to have bactericidal activity only in the first eight hours of treatment, with regrowth thereafter using time-kill curve analysis [12]. However, combination therapy with tigecycline and colistin was found to have bactericidal and synergistic activity at 24 hours. We have encountered similar results with different polymyxin B combination therapy, in which bactericidal activity was noted throughout the dosing intervals based on consistent trough bactericidal titers found in our patients. 

In a recent study by Urban et al. combination therapy of polymyxin B, doripenem, and rifampin showed bactericidal activity using time-kill curve against two strains of KPC-producing KP [13]. Combination of any two of the three antibiotics tested, showed no bactericidal activity against the isolates. Furthermore, combination therapy seems to have a role in isolates with decreased polymyxins susceptibility [14]. In this study, the serum of all three patients contained polymixin B and an additional antibiotic, so we cannot assume that the bactericidal activity seen in the combination therapy was not solely due to polymyxin B. However, the bactericidal activity noted against polymyxin-resistant KP isolates in our study indicates that there might be some synergism. SBT results from study case number 3 showed that the combination of polymyxin B, rifampin, and doripenem had bactericidal activity despite showing resistance against each individual agent. 

Tigecycline has been used in the treatment of carbapenem-resistant organisms; however, its low serum levels are a concern in bacteremic patients [15], thus combination therapy may be beneficial in treatment of KPC KP bacteremia. Humphries et al. were able to treat colistin-resistant KPC-positive KP bacteremia with tigecycline and colistin [15]. We also obtained microbiological cure with a combination of tigecycline and polymyxin B, and we believe that tigecycline can be an important addition in the treatment of KPC-producing KP bacteremia if used in combination therapy. Doripenem is the newest carbapenem available in the market, and was used in combination therapy in two of our patients. In a recent animal study, doripenem given as 4-hour infusion was noted to produce modest CFU reduction (1 log reduction) of KPC-producing KP in the presence of white blood cells, irrespective of the doripenem MIC (range from 4 to 32 *μ*g/dL) [16]. The results did not showed any bactericidal activity but the authors concluded that in immunocompetent patients, doripenem might have a role, in combination therapy, in the treatment of KPC-producing KP infections.

 Most studies of combination therapy for KPC-producing KP infections had been done *in vitro* with the use of time-kill curves or checkerboards which do not take into account the pharmacokinetics of the antibiotics used. Polymyxins have been in clinical use for over four decades but variable pharmacokinetics of this class of antibiotics has been reported [17]. SBT has the theoretical advantage of assessing the pharmacokinetic and pharmacodynamics of the antimicrobials in an individual patient [18, 19]. In a case report of a vancomycin-resistant *Enterococcus faecium* endocarditis treated with daptomycin, clinical failure was predicted by the SBT and not the MIC [20]. Low free daptomycin levels were found due to its high protein affinity which was not assessed by the MIC. 

SBT has been used to evaluate antibiotic interactions in combination therapy [21–23] and to predict clinical outcome. Martino et al. reported that peak and trough SBT of >1 : 8 of beta-lactam-aminoglycosides combination therapy predicted clinical success in neutropenic patients with Gram-negative septicemia [24]. Similar peak SBT of >1 : 8 predicted clinical success in endocarditis [25] and in oncology patients with septicemia and wound infections [18]. Patients who receive combination therapy have higher SBT and higher cure rate than patients who received only single-agent therapy, suggesting the possibility of synergism [18]. However, if SBT of ≥1 : 16 are obtained, this correlates with good clinical outcome despite the use of single or combination antimicrobial therapy [24]. SBT has also been used in the management of osteomyelitis [26], acute pulmonary exacerbation in cystic fibrosis patients [27], and treatment of infections caused by organisms with reduced antimicrobial susceptibility [28, 29]. Variability of the SBT among different laboratories was a limiting factor for the acceptance of this test for clinical use [30, 31]. Timing of the sampling of the specimens relative to the antibiotic dose, variation of the dilution of serum, size of bacterial inoculum, culture media used, and time of incubation were among the factors accounting for intra- and interlaboratory variability. However, standardized methods for the SBT determination have been published and endorsed by national guidelines [6]. 

Microbiological success was obtained in our three cases of KPC producing KP bacteremia with different polymyxin B containing combination therapies. Clinical success was seen in two of the patients with the third patient expiring from other medical comorbidities. As the MIC for polymixin B increases, the SBT decreases for the three antibiotic combinations used highlighting the importance of polymixin B for increased bactericidal activity with combination therapy for the treatment of KPC-producing KP infections. Therefore, according to the results of our study, combination therapy with polymixin B-containing therapies may be an option in the treatment of bacteremia caused by KPC-producing KP. Additionally, SBT may have a role in the guidance of treatment of patients with carbapenem-resistant KP infections and other multidrug-resistant Gram-negative bacteria.

## Figures and Tables

**Table 1:** Peak and trough serum inhibitory/bactericidal titers.

		Peak		Trough		MIC (*μ*g/mL) by Etest			
	Isolate	SIT	SBT	SIT	SBT	DOR	PO	TG	RIF
Case 1	Patient	1 : 32	1 : 32	1 : 16	1 : 16	>32	0.5		
	NYHQ KP	1 : 4	1 : 4	1 : 4	1 : 4	3	6		
	*E. coli *	>1 : 1024	>1 : 1024	>1 : 1024	>1 : 1024	0.016	0.5	0.125	12
	KP SHV-18	1 : 256	1 : 256	1 : 128	1 : 128	0.064	1	ND	>32

Case 2	Patient	1 : 32	1 : 32	1 : 32	1 : 16	16	2	3-4	

Case 3	Patient	1 : 8	1 : 8	1 : 8	1 : 8	3	3		>32
	NYHQ KP	1 : 8	1 : 8	1 : 8	1 : 8	>32	32		>32

SIT: serum inhibitory titers; SBT: serum bactericidal titers; MIC: minimal inhibitory concentration; DOR: doripenem; PO: polymyxin B; TG: tigecycline; RIF: rifampin; KP: *Klebsiella pneumoniae*; ND: not done.
